# Distinguishing exercise intolerance in early‐stage pulmonary hypertension with invasive exercise hemodynamics: Rest *V*_E_/*V*CO_2_ and ETCO_2_ identify pulmonary vascular disease

**DOI:** 10.1002/clc.23831

**Published:** 2022-04-14

**Authors:** Farhan Raza, Naga Dharmavaram, Timothy Hess, Ravi Dhingra, James Runo, Amy Chybowski, Callyn Kozitza, Supria Batra, Evelyn M. Horn, Naomi Chesler, Marlowe Eldridge

**Affiliations:** ^1^ Department of Medicine‐Division of Cardiology University of Wisconsin‐Madison Madison Wisconsin USA; ^2^ Department of Medicine‐Division of Pulmonary and Critical Care University of Wisconsin‐Madison Madison Wisconsin USA; ^3^ Department of Biomedical Engineering University of Wisconsin‐Madison Madison Wisconsin USA; ^4^ Department of Medicine‐Division of Cardiology Weill Cornell Medicine New York New York USA; ^5^ University of California‐Irvine Edwards Lifesciences Foundation Cardiovascular Innovation and Research Center and Department of Biomedical Engineering Irvine California USA; ^6^ Department of Pediatrics University of Wisconsin‐Madison Madison Wisconsin USA

**Keywords:** cardiopulmonary exercise test, ETCO_2_, invasive exercise hemodynamics, pulmonary hypertension, pulmonary vascular disease, *V*_E_/*V*CO_2_

## Abstract

**Background:**

Among subjects with exercise intolerance and suspected early‐stage pulmonary hypertension (PH), early identification of pulmonary vascular disease (PVD) with noninvasive methods is essential for prompt PH management.

**Hypothesis:**

Rest gas exchange parameters (minute ventilation to carbon dioxide production ratio: *V*_E_/*V*CO_2_ and end‐tidal carbon dioxide: ETCO_2_) can identify PVD in early‐stage PH.

**Methods:**

We conducted a retrospective review of 55 subjects with early‐stage PH (per echocardiogram), undergoing invasive exercise hemodynamics with cardiopulmonary exercise test to distinguish exercise intolerance mechanisms. Based on the rest and exercise hemodynamics, three distinct phenotypes were defined: (1) PVD, (2) pulmonary venous hypertension, and (3) noncardiac dyspnea (no rest or exercise PH). For all tests, *p < .05 was considered statistically significant.

**Results:**

The mean age was 63.3 ± 13.4 years (53% female). In the overall cohort, higher rest *V*_E_/*V*CO_2_ and lower rest ETCO_2_ (mm Hg) correlated with high rest and exercise pulmonary vascular resistance (PVR) (*r* ~ 0.5–0.6*). On receiver‐operating characteristic analysis to predict PVD (vs. non‐PVD) subjects with noninvasive metrics, area under the curve for pulmonary artery systolic pressure (echocardiogram) = 0.53, rest *V*_E_/*V*CO_2_ = 0.70* and ETCO_2_ = 0.73*. Based on this, optimal thresholds of rest *V*_E_/*V*CO_2_ > 40 mm Hg and rest ETCO_2_ < 30 mm Hg were applied to the overall cohort. Subjects with both abnormal gas exchange parameters (*n* = 12, vs. both normal parameters, *n* = 19) had an exercise PVR 5.2 ± 2.6* (vs. 1.9 ± 1.2), mPAP/CO slope with exercise 10.2 ± 6.0* (vs. 2.9 ± 2.0), and none included subjects from the noncardiac dyspnea group.

**Conclusions:**

In a broad cohort of subjects with suspected early‐stage PH, referred for invasive exercise testing to distinguish mechanisms of exercise intolerance, rest gas exchange parameters (*V*_E_/*V*CO_2_ > 40 mm Hg and ETCO_2_ < 30 mm Hg) identify PVD.

**Abbreviations**
CO: cardiac output
CPET: cardiopulmonary exercise test
ETCO_2_
: end‐tidal carbon dioxide pressure
mPAP: mean pulmonary artery pressure
PVR: pulmonary vascular resistance
*V*_E_/*V*CO_2_
: minute ventilation to carbon dioxide production ratio

## 1 INTRODUCTION

Pulmonary hypertension (PH) diagnosis is based on invasive hemodynamics and is defined by mean pulmonary artery (PA) pressure (mPAP) ≥20 mm Hg at rest.^1^ While a broad range of pathologies lead to PH,^2^, ^3^, ^4^, ^5^ a small proportion of patients with precapillary PH with normal left‐sided filling pressures (aka. pulmonary vascular disease: PVD) benefit from pulmonary vasodilator therapy.^1^, ^6^ Hence, prompt recognition of PVD with noninvasive clinical tools and selective use of invasive hemodynamic testing is essential in the clinical care of a patient with dyspnea and suspected PH.^7^, ^8^, ^9^, ^10^ While early diagnosis of PH and predominant phenotype of PH can be established with exercise invasive hemodynamics^11^, ^12^, ^13^, ^14^ and simultaneous expired gas analysis (cardiopulmonary exercise testing: CPET) at expert centers,^15^, ^16^, ^17^ the noninvasive CPET facilities are more widely available. Moreover, in patients unable to undergo a maximal exercise test and who would benefit from an ambulatory test to identify PVD, the role of gas exchange parameters at rest needs to be explored.^18^, ^19^

In this study, we report on the invasive exercise cardiopulmonary hemodynamics and simultaneous expired gas analysis from a broad cohort of subjects with exercise intolerance and dyspnea (suspected due to PH at rest and/or with exercise). Our goal is to identify significant patterns of gas exchange parameters among different phenotypes of subjects with dyspnea and improve upon noninvasive rest diagnostic tools for early identification of PVD.

## 2 METHODS

### 2.1 Study design and cohort

We conducted a retrospective review of a cohort of subjects presenting to the University of Wisconsin (UW)‐Madison PH clinic for exercise intolerance and dyspnea with New York Heart Association Class II–III. With a significant burden of subjective symptoms, and yet subclinical‐mild PH and relatively preserved right ventricular (RV) function on echocardiogram, the subjects were referred for an invasive exercise hemodynamic study with CPET to discriminate mechanisms of exercise intolerance. These mechanisms may include chronotropic incompetence, poor pulmonary compliance, rapid rise of intracardiac filling pressure, valvular heart disease, and/or peripheral muscular limitations.^20^ Early‐stage PH and referral for exercise hemodynamic study was based on low‐intermediate probability of PH per ESC/ERS guidelines.^6^ We evaluated 55 consecutive subjects over a 1‐year period. All subjects underwent clinical evaluation, six‐minute walk test, echocardiogram, and pulmonary function testing. Other appropriate diagnostic PH workup (ventilation–perfusion and noncontrast chest computed tomography scans) was performed as indicated. Exclusion criteria for this study were as follows left ventricular ejection fraction <50%, supplemental oxygen use, oxygen saturation (SpO_2_) <95% room air on six‐minute walk test, primary lung disease with forced expiratory volume in 1 second (FEV1) < 60% predicted, primary tricuspid valve disease, and age >80 years.

### 2.2 Invasive CPET

**Figure 1:**
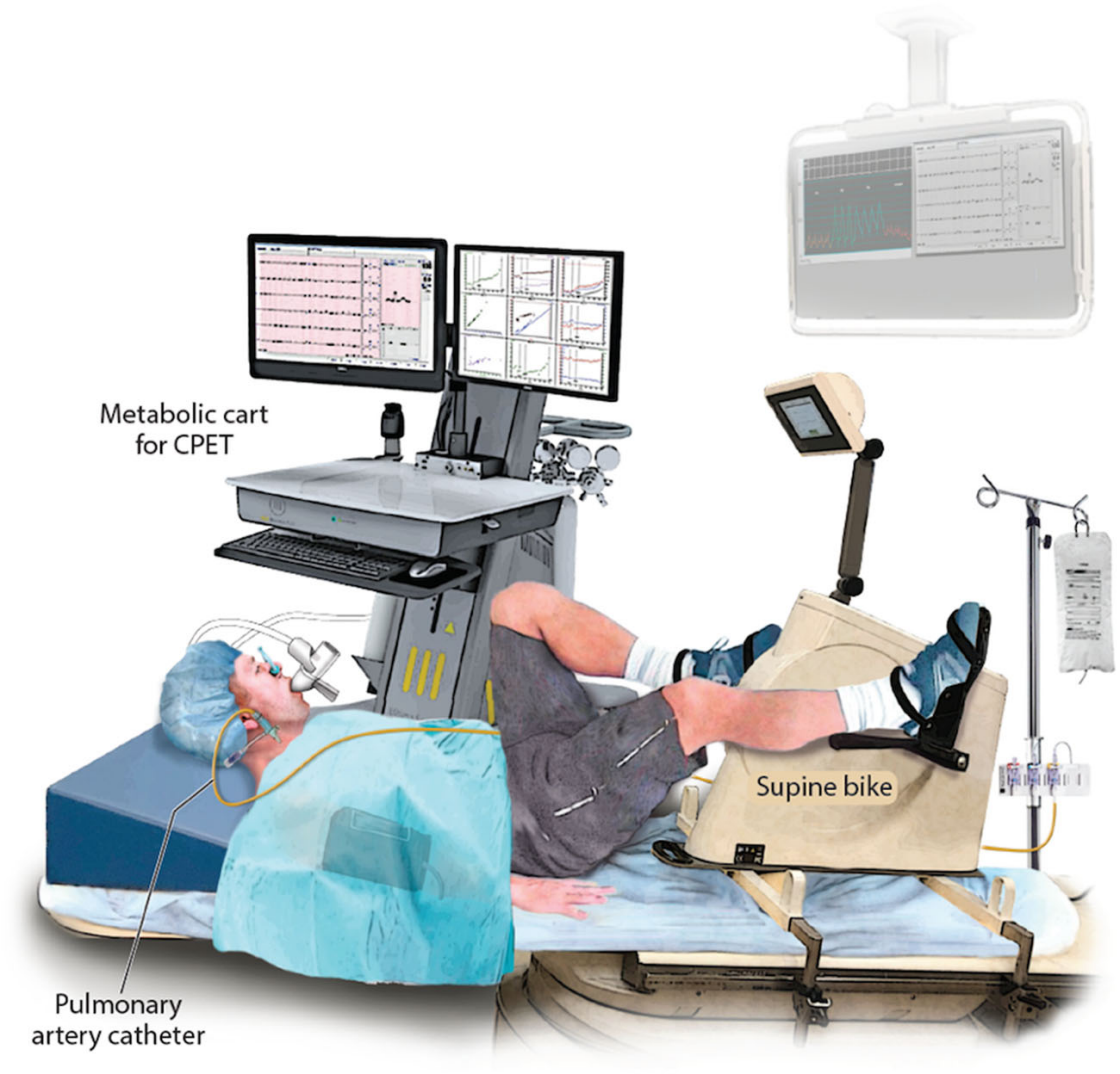
Representative figure of invasive exercise hemodynamics with cardiopulmonary exercise testing. CPET, cardiopulmonary exercise test.

All subjects underwent hemodynamic evaluation with a right heart catheterization (RHC) and simultaneous expired gas analysis at rest, during exercise, and at recovery, as previously described^21^ (Figure 1). Pulmonary hemodynamic measurements were obtained with a balloon‐tipped, double‐lumen, fluid‐filled 7 Fr PA catheter via an internal jugular vein approach. Cardiac output (CO) was measured via both direct Fick principle and thermodilution methods. After the fluid‐filled catheter placement in the PA, a nose clip and a mouthpiece with a saliva trap were placed to confirm an air leak‐free system. The mouthpiece was connected to an umbilical adapter and further connected to Metabolic Ultima™ CardiO2® gas exchange analysis system (MedGraphics) for a breath‐by‐breath analysis. After connection with the metabolic cart, subjects were observed for 5 minutes to achieve a steady rest state, and after reaching equilibrium, resting gas exchange parameters are recorded. A PA mixed venous sample was drawn at the same time for direct Fick CO at rest.

After rest hemodynamics were recorded, the legs were placed on the pedals of a stationary supine ergometer and the subject's upper body was lifted to a 45° incline with a firm wedge‐shaped pillow. The transducer was reequilibrated at the level of the left atrium. After 5 minutes, repeat hemodynamics were recorded. Subsequently, subjects were exercised on a semirecumbent cycle ergometer at 60 rpm with continuous electrocardiographic monitoring. Exercise was started with unloaded peddling (0 W) for 2 minutes and then resistance increased on an incremental 10–20 W/min protocol. Mean PA pressures and mean PA wedge pressures (PAWPs) were recorded every 20–25 W till peak exercise to trend the pressures, which increased confidence in peak‐exercise pressure readings. Near peak exercise (limited by symptoms), a mixed venous sample from the distal PA port was taken (while the subjects were still exercising), VO_2_ was noted (for direct Fick CO for peak exercise), and hemodynamic parameters (PA pressure, PAWP, RV, and right atrial pressures) were recorded with reported mean values (averaged over 3–5 respiratory cycles). The PA catheter and venous sheath were removed at the end of the study. A radial arterial line was not used.

### 2.3 Statistical analysis

Categorical variables are summarized as counts and percentages and differences were assessed using either the *χ* ^2^ statistic or Fisher exact test, as appropriate. Continuous data are presented as mean ± SD and statistical significance was tested using Student *t* test and analysis of variance (ANOVA) test, as appropriate. However, for non‐normally distributed data, Wilcoxon rank‐sum and Kruskal–Wallis tests were used. Post hoc pairwise comparisons were performed among different groups. Simple linear regression analysis was used to examine correlations between invasive hemodynamics and expired gas exchange (CPET) parameters. Receiver‐operating characteristic (ROC) analysis was performed to assess the area under the curve (AUC) using gas exchange parameters at rest and after exercise to predict PVD. Tests of the AUC evaluated the hypothesis that the AUC was different from 0.50. The optimal thresholds for rest gas exchange parameters were identified with values that maximized the sum of sensitivity plus specificity. For all tests, *p* values less than .05 were considered statistically significant. Data were analyzed in SPSS v26.0 (IBM Corp.) and R (version 4.0.3).

## 3 RESULTS

### 3.1 Hemodynamic profiling into PVD, pulmonary venous hypertension (PVH), and noncardiac dyspnea (NCD) groups

After the invasive exercise hemodynamics, a final diagnosis of a predominant World Health Organization (WHO) group was established. The distribution of subjects in different PH WHO groups was (*n*, %): WHO Group I (10, 18%), WHO Group II (33, 60%), WHO Group III (2, 4%), and WHO Group IV (2, 4%). Eight subjects (14%) had no rest or exercise PH. The overall cohort was classified into three groups based on resting hemodynamics, and also taking exercise hemodynamics into the account:

1. PVD (*n* = 14): PVD group had rest mPAP ≥ 20 mm Hg, PAWP ≤15 mm Hg, and pulmonary vascular resistance (PVR) ≥3 Woods units (WU). With exercise, mPAP/CO slope was ≥3 mm Hg/L/min,^17^ peak exercise transpulmonary resistance (TPR) ≥ 3 mm Hg/L/min,^22^ PAWP/CO slope with exercise <2 mm Hg/L/min, and mPAP > 30 mm Hg.^23^
2. PVH (*n* = 33): PVH had rest mPAP ≥ 20 mm Hg and PAWP > 15 mm Hg. With exercise, mPAP/CO slope was ≥3 mm Hg/L/min, TPR ≥ 3 mm Hg/L min,^22^ PAWP/CO slope ≥ 2 mm Hg/L/min,^24^ and mPAP > 30 mm Hg. Two subjects with rest mPAP < 20 mm Hg and PAWP ≤ 15 mm Hg were classified into the PVH group due to their exercise hemodynamics (exercise mPAP = 37 ± 7 mm Hg, PAWP = 24 ± 2 mm Hg, mPAP/CO = 4.7 ± 1.0 mm Hg/L/min, PAWP/CO = 2.9 ± 0.6 mm Hg/L/min, and TPR = 4.1 ± 0.8 mm Hg/L/min).
3. The NCD (*n* = 8) group had rest mPAP < 20 mm Hg, PAWP ≤ 15 mm Hg, and PVR < 3 WU. With exercise, mPAP/CO slope is <3 mm Hg/L/min and TPR is <3 mm Hg/L/min. Three subjects with mPAP 20–24 mm Hg were classified into this group due to: rest PAWP < 15 mm Hg, rest PVR < 3 WU, and they did not meet exercise PH criteria (as mentioned above: mPAP/CO slope and exercise TPR).

The PVD group (*n* = 14) comprised of a combination of WHO Group I/III/IV, the PVH group (*n* = 33) comprised of a WHO Group II, and the NCD group comprised of subjects with no rest or exercise PH (*n* = 8). The distribution of postcapillary and precapillary phenotypes was identical to the incidence reported in PH epidemiological studies.^25^, ^26^ Among the subjects diagnosed with PH (*n* = 47), the incidence of postcapillary PH phenotype (WHO Group II) was 70% (*n* = 33), and 30% for precapillary PH phenotype (*n* = 14). All PVD with exercise subjects were started on PH vasodilator therapy, while none of the PVH or NCD subjects was offered PH therapy.^8^, ^17^, ^18^, ^19^, ^20^, ^21^

### 3.2 PVD, PVH, and NCD groups: Clinical characteristics

The clinical and imaging profile of three groups is represented in Table 1. The PVD group had a higher incidence of scleroderma and the lowest diffusion capacity of carbon monoxide on pulmonary function tests. The two subjects in WHO Group III had a primary diagnosis of sleep‐disordered breathing. As mentioned previously, chronic obstructive pulmonary disease patients with FEV1 < 60% were excluded from the study. The PVH group had significant comorbidities (hypertension, atrial fibrillation, obstructive sleep apnea, and obesity), lowest six‐minute walk distance, and the highest brain natriuretic peptide (BNP) levels. The NCD group had patients who were younger, less burden of comorbidities, and near‐normal BNP level.

**Table 1:** Clinical and echocardiographic features of three groups: PVD, PVH, and NCD

Parameters	PVD (*n* = 14)	PVH (*n* = 33)	NCD (*n* = 8)	*p* Value
Clinical				
Age (years)	67 ± 10	65 ± 13	51 ± 14	.01^†^ ^,^ ^‡^
Female sex, *n* (%)	7 (50%)	16 (48%)	6 (75%)	.39
Comorbidities, *n* (%)				
DM	4 (28%)	7 (21%)	1 (8%)	.67
HTN	9 (64%)	28 (85%)	3 (37%)	.02
CKD	2 (14%)	13 (39%)	1 (12%)	.12
Afib	3 (21%)	16 (48%)	1 (12%)	.07
OSA	4 (29%)	13 (39%)	2 (25%)	.64
CAD	3 (21%)	13 (39%)	0 (0%)	.08
COPD	2 (14%)	6 (18%)	1 (12%)	.90
PE history	3 (21%)	5 (15%)	0 (0%)	.39
Scleroderma	6 (43%)	1 (3%)	1 (12%)	.002*
BMI (kg/m^2^)	25.7 ± 4.7	33.2 ± 8.0	28.1 ± 2.7	.01
6MWD (m)	362 ± 80	251 ± 103	476 ± 47	<.001
BNP (pg/ml)	192 ± 207	294 ± 273	51 ± 51	.24
DLCO (%)	56 ± 15	68 ± 16	85 ± 20	.01
Echocardiogram^a^				
LVEF (%)	58 ± 9	57 ± 11	59 ± 8	.85
TAPSE (cm)	2.0 ± 4.8	2.0 ± 6.3	2.0 ± 0.7	.96
Lateral *E*/*e*′	7.5 ± 3.0	13.7 ± 6.9	7.9 ± 2.3	.03
LAVI (ml/m^2^)	34 ± 16	43 ± 12	22 ± 2	.004
PASP (mm Hg)	45 ± 13	50 ± 19	24 ± 6	.06

Abbreviations: Afib, atrial fibrillation; BMI, body mass index; BNP, brain natriuretic peptide; CKD, chronic kidney disease; CAD, coronary artery disease; COPD, chronic obstructive pulmonary disease; DM, diabetes mellitus; DLCO, diffusion capacity of carbon monoxide; *E*/*E*′, ratio of early diastolic velocity of mitral inflow to early diastolic tissue Doppler velocity of the lateral mitral annulus; HTN, hypertension; LAVI, left atrial volume index; LVEF, left ventricular ejection fraction; 6MWD, six‐minute walk distance; NCD, noncardiac dyspnea; OSA, obstructive sleep apnea; PASP, pulmonary artery systolic pressure; PE, pulmonary embolism; PVD, pulmonary vascular disease; PVH, pulmonary venous hypertension; TAPSE, tricuspid annular plane excursion.

^a^ Echo was performed within 1 month after heart catheterization.

For pairwise comparisons, *PVD versus PVH, ^†^PVD versus NCD, ^‡^PVH versus NCD.

### 3.3 PVD, PVH, and NCD groups: Hemodynamic and metabolic profile

The hemodynamic profile of the three groups is summarized in Table 2. The peak PAWP ranged from 22 to 38 mm Hg in the PVH group, while it ranged from 15 to 22 mm Hg in the PVD group. Among the three PVD subjects with PAWP ≥ 20 mm Hg (22 mm Hg among all three subjects), the PAWP/CO slope was 1.5 ± 0.4 (mm Hg/L/min) and the PAWL (peak exercise PAWP‐indexed to peak workload in W/body weight [kg]) was 17.1 ± 3.8 (mm Hg/W/kg), consistent with the absence of postcapillary PH.^24^, ^27^

**Table 2:** Hemodynamic and metabolic features of three groups: PVD, PVH, and NCD

Parameters	PVD (*n* = 14)	PVH (*n* = 33)	NCD (*n* = 8)	*p* Value
Rest				
mPAP^a^ (mm Hg)	37 ± 8	33 ± 9	21 ± 4	.001^†^ ^,^ ^‡^
PAWP (mm Hg)	13 ± 2	18 ± 4	12 ± 2	<.001* ^,^ ^‡^
Direct Fick CO (L/min)	4.3 ± 1.0	5.1 ± 1.2	5.7 ± 0.9	.015^†^
Direct Fick CI (L/min/m^2^)	2.4 ± 0.5	2.5 ± 0.5	3.0 ± 0.5	.017^†^ ^,^ ^‡^
SvO_2_ (%)	65 ± 5	64 ± 6	72 ± 7	.003^†^ ^,^ ^‡^
DPG (mm Hg)	11 ± 7	6 ± 5	2 ± 2	.002* ^,^ ^†^
PVR (WU)	5.7 ± 2.1	3.0 ± 1.8	1.4 ± 0.7	<.001* ^,^ ^†^
PCa (ml/mm Hg)	2.1 ± 0.9	3.9 ± 1.9	5.4 ± 1.4	<.001* ^,^ ^†^
Peak exercise				
mPAP (mm Hg)	55 ± 9	51 ± 12	29 ± 3	<.001^†^ ^,^ ^‡^
PAWP (mm Hg)	19 ± 3	31 ± 7	18 ± 2	<.001^†^ ^,^ ^‡^
Direct Fick CO (L/min)	7.4 ± 1.8	7.8 ± 2.3	12.7 ± 4.6	<.001^†^ ^,^ ^‡^
Direct Fick CI (L/min/m^2^)	4.1 ± 0.9	3.9 ± 0.9	6.7 ± 2.4	<.001^†^ ^,^ ^‡^
SvO_2_ (%)	38 ± 10	30 ± 10	40 ± 8	.01^‡^
DPG (mm Hg)	16 ± 7	6 ± 6	4 ± 4	<.001* ^,^ ^†^
PVR (WU)	5.1 ± 2.2	2.9 ± 2.2	1.1 ± 0.6	<.001* ^,^ ^†^
PCa (ml/mm Hg)	1.6 ± 0.6	2.4 ± 1.1	4.3 ± 1.9	<.001^†^ ^,^ ^‡^
Delta with exercise				
mPAP/CO slope (mm Hg/L/min)	12.9 ± 2.9	9.6 ± 5.3	1.8 ± 1.0	.03^†^ ^,^ ^‡^
PAWP/CO slope (mm Hg/L/min)	1.4 ± 0.5	11.5 ± 8.2	1.0 ± 0.6	.02* ^,^ ^‡^
PAWL^b^ (mm Hg/W/kg)	18.5 ± 5.6	39.5 ± 18.7	14.8 ± 8.5	<.001* ^,^ ^‡^
Peak‐exercise TPR (mm Hg/L/min)	8.1 ± 3.4	7.3 ± 3.8	1.7 ± 0.4	<.001^†^ ^,^ ^‡^
CPET: Peak workload parameters				
Peak workload (W)	55 ± 31	57 ± 29	125 ± 66	<.001^†^ ^,^ ^‡^
Indexed workload^a^	0.74 ± 0.38	0.64 ± 0.36	1.58 ± 0.81	<.001^†^ ^,^ ^‡^
Peak *V*O_2_ (mL/kg/min)	10.3 ± 3.2	9.2 ± 2.4	17.2 ± 8.2	<.001^†^ ^,^ ^‡^
Peak O_2_ pulse (ml/min)	11.7 ± 13.2	10.6 ± 7.3	10.6 ± 4.4	.92
Peak heart rate	94 ± 15	93 ± 20	125 ± 22	<.001^†^ ^,^ ^‡^
RER	1.00 ± 0.09	1.00 ± 0.11	1.12 ± 0.04	.02^†^ ^,^ ^‡^
CPET: Gas exchange parameters				
Rest *V*_E_/*V*CO_2_	46 ± 8	42 ± 6	37 ± 3	.03^†^
Exercise *V*_E_/*V*CO_2_	44 ± 9	39 ± 8	32 ± 3	.003^†^ ^,^ ^‡^
Rest ETCO_2_ (mm Hg)	29 ± 5	32 ± 4	35 ± 3	.017^†^
Exercise ETCO_2_ (mm Hg)	28 ± 6	31 ± 5	38 ± 3	<.001^†^ ^,^ ^‡^

Abbreviations: CO, cardiac output; CI, cardiac index; DPG, diastolic pulmonary gradient; ETCO_2_, end‐tidal carbon dioxide pressure; mPAP, mean pulmonary artery pressure; NCD, noncardiac dyspnea; O_2_ pulse, *V*O_2_/heart rate; PAWL, peak exercise pulmonary artery wedge pressure/indexed peak workload (peak W/weight [kg]); PAWP, pulmonary artery wedge pressure; PCa, pulmonary compliance; PVD, pulmonary vascular disease; PVH, pulmonary venous hypertension; PVR, pulmonary vascular resistance; RER, respiratory exchange ratio; SvO_2_, mixed venous sample saturation; TPR, transpulmonary resistance (mean PAP/CO); *V*_E_/*V*CO_2_, minute ventilation to carbon dioxide production ratio; *V*O_2_, oxygen consumption.

^a^ Rest mPAP range: PVD = 26–58; PVH = 15–52 (among two subjects had rest mPAP < 20 and PAWP < 15, at peak exercise = mPAP 37 ± 7, PAWP 24 ± 2, mPAP/CO 4.7 ± 1.0, PAWP/CO 2.9 ± 0.6 TPR 4.1 ± 0.8 mm Hg/L/min); NCD = 18–23.

^b^ Indexed workload (W/kg) <0.5 excluded (PVD: 10/14; PVH: 20/33; NCD: 8/8).

For pairwise comparisons, *PVD versus PVH, ^†^PVD versus NCD, and ^‡^PVH versus NCD.

The augmentation of CO with exercise was significantly and equally impaired for both PVD and PVH groups (<80%), in comparison to the NCD group (>120%). This was contributed equally by both stroke volume and heart rate. The net peripheral oxygen extraction (*C*[*a* − v]O_2_ [ml/dl]) from rest to exercise was similar among all the three groups: PVD (5.9–>11.2), PVH (5.6–>10.9) and NCD (4.5–>10.4). Among all groups, PVR decreased somewhat with exercise. The peak workload was also equally impaired (peak watts and VO_2_) in PVD and PVH groups, in comparison to the NCD. Anaerobic threshold (respiratory exchange ratio at peak exercise >1.05) was achieved by 50% of PVD (7/14), 39% of PVH (13/33), and 100% of NCD (8/8) subjects.

The gas exchange parameters (minute ventilation to carbon dioxide production ratio: *V*_E_/*V*CO_2_ and end‐tidal carbon dioxide: ETCO_2_) were incrementally abnormal in this order: PVD > PVH > NCD. The differentiable response of gas exchange parameters between the three groups was significant at rest and changed in a similar fashion with exercise among the three groups. The breathing reserve was normal (>20%) and SpO_2_ remained ≥95% (on pretest six‐minute walk test and during invasive exercise hemodynamic study) in all subjects in the whole cohort.

### 3.4 Rest gas exchange parameters (*V*_E_/*V*CO_2_ and ETCO_2_) and hemodynamic profiles

**Figure 2:**
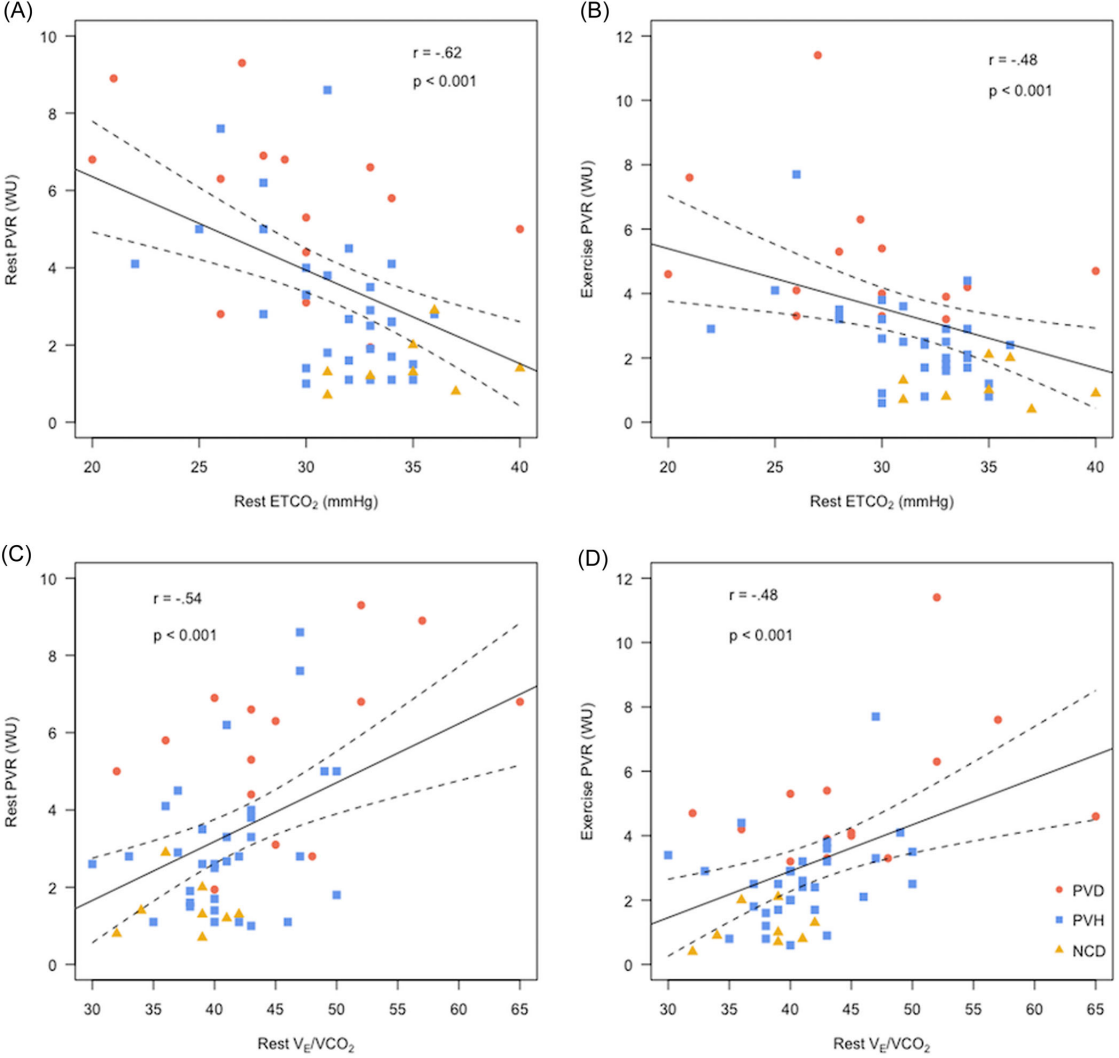
Correlation of rest ETCO_2_ (A&B) and *V*_E_/*V*CO_2_ (C&D) with rest and exercise PVR. ETCO_2_, end‐tidal carbon dioxide pressure; PVR, pulmonary vascular resistance; *V*_E_/*V*CO_2_, minute ventilation to carbon dioxide production ratio.

To investigate the clinical utility of rest gas exchange parameters in recognizing PVD in subjects with dyspnea, we identified a moderate correlation of rest *V*_E_/*V*CO_2_ and ETCO_2_ with rest PVR and exercise PVR (Figure 2). On ROC analysis to predict PVD (vs. non‐PVD) phenotype, the AUC was significant for both rest *V*_E_/*V*CO_2_ (0.70, *p* < .01) and ETCO_2_ (0.73, *p* < .01). In comparison to the commonly utilized noninvasive parameter of echocardiogram based pulmonary artery systolic pressure (PASP) to diagnose PH (AUC = 0.53, *p* = .08), rest *V*_E_/*V*CO_2_ and ETCO_2_ performed better in predicting PVD. Of note, AUC was similar for exercise *V*_E_/*V*CO_2_ (0.76, *p* < .01) and ETCO_2_ (0.73, *p* < .01) in predicting PVD. Correlations of rest *V*_E_/*V*CO_2_ and ETCO_2_ with exercise transpulmonary resistance (TPR) were weaker (*r* = .36, *p* = .007 for *V*_E_/*V*CO_2_ and *r* = .25, *p* = .06 for ETCO_2_).

### 3.5 Utility of rest gas exchange parameters for clinical screening

**Figure 3:**
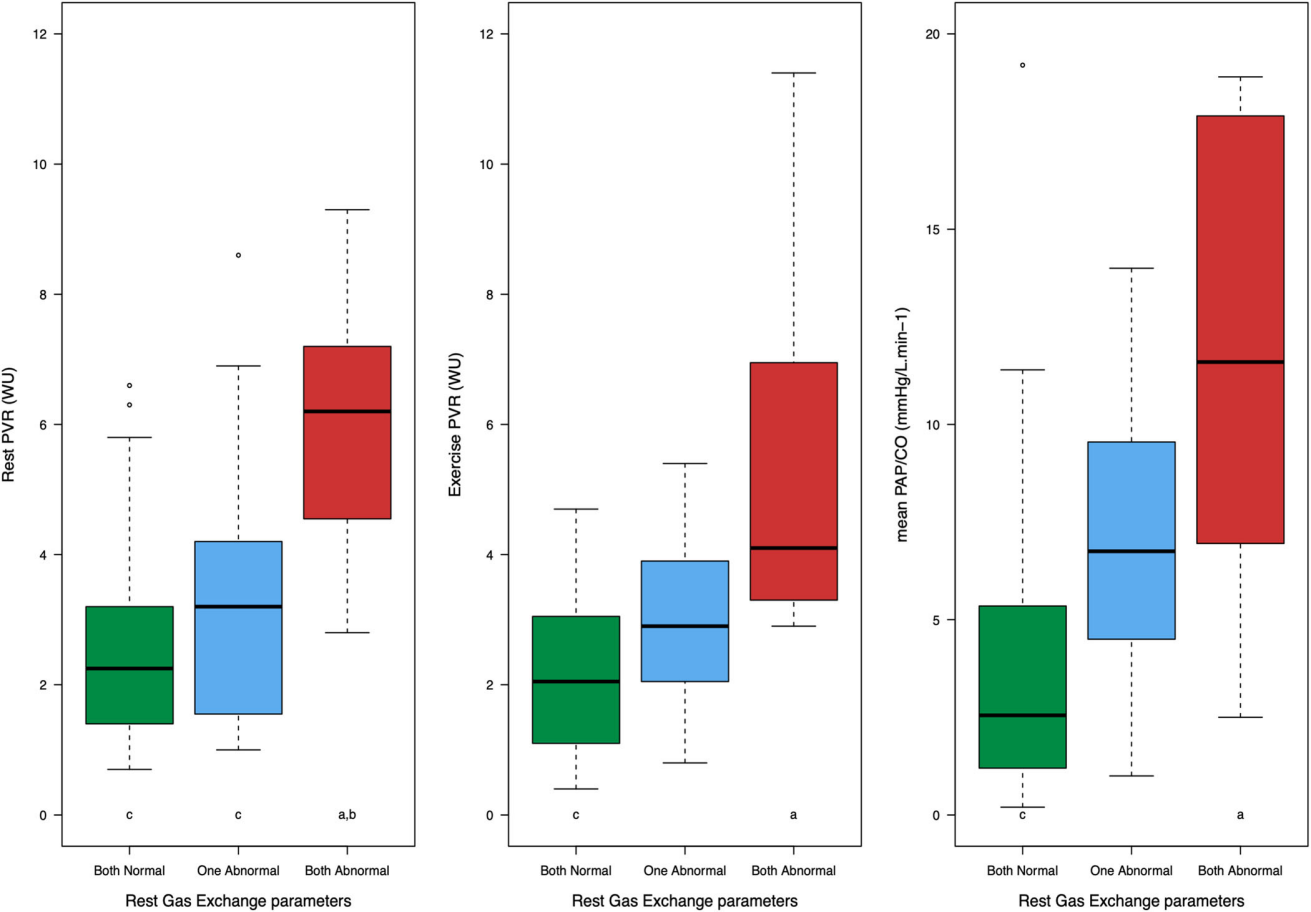
Distribution of rest gas exchange parameters among the overall cohort and hemodynamic profile. Abnormal thresholds for rest ETCO_2_ (<30 mm Hg) and *V*_E_/*V*CO_2_ (>40 mm Hg) Both normal: green (*n* = 26), one abnormal: blue (*n* = 17), both abnormal: red (*n* = 12). ANOVA *p* < .001 for all three analyses. For pairwise comparisons, both normal (green) = a, one abnormal (blue) = b, and both abnormal (red) = c. ANOVA, analysis of variance; CO, cardiac output; ETCO_2_, end‐tidal carbon dioxide pressure; mPAP, mean pulmonary artery pressure; PET, cardiopulmonary exercise test; PVR, pulmonary vascular resistance; *V*_E_/*V*CO_2_, minute ventilation to carbon dioxide production ratio.

On ROC analysis to predict PVD phenotype with exercise, we identified optimal thresholds for rest *V*_E_/*V*CO_2_ > 40 mm Hg and ETCO_2_ < 30 mm Hg. These thresholds are consistent with historically reported thresholds for significant PH.^27^ Applying these thresholds for rest *V*_E_/*V*CO_2_ and ETCO_2_ to the overall cohort, we identified 26 subjects with both normal, 17 subjects with one abnormal, and 12 subjects with both abnormal. The hemodynamic profile of this distribution is shown in Figure 3. Of note, none of the subjects in the NCD group had two abnormal rest gas exchange parameters.

### 3.6 Validation cohort

To validate the clinical utility of rest *V*_E_/*V*CO_2_ > 40 mm Hg and ETCO_2_ < 30 mm Hg in identifying PVD, we identified a cohort of subjects evaluated at Weill Cornell Medical Center for dyspnea and suspected PH. During a 6‐month time period, we identified 25 consecutive subjects who underwent a noninvasive CPET on a cycle ergometer followed closely by a resting RHC (median interval between the two studies: 21 days; interquartile range: 7, 36). The mean age for this cohort was 57.7 ± 15.5% and 40% were female. We applied the thresholds for rest *V*_E_/*V*CO_2_ > 40 mm Hg and ETCO_2_ < 30 mm Hg as abnormal rest gas exchange parameters.

Among the overall validation cohort, nine subjects with both abnormal rest gas exchange parameters had a PVR of 7.2 ± 2.5 WU (mPAP = 40 ± 8 mm Hg; PAWP = 9 ± 4 mm Hg; CO = 4.7 ± 2.1 L/min). Nine subjects with one abnormal rest gas exchange parameter had a PVR of 4.2 ± 1.4 WU (mPAP = 34 ± 7 mm Hg; PAWP = 14 ± 6 mm Hg; CO = 5.1 ± 2.8 L/min). Seven subjects with both normal rest gas exchange parameters had a PVR of 2.5 ± 1.5 WU (mPAP = 29 ± 12 mm Hg; PAWP = 16 ± 6 mm Hg; CO = 6.2 ± 2.3 L/min).

## 4 DISCUSSION

In this study, we report on the utility of rest gas exchange parameters (*V*_E_/*V*CO_2_ and ETCO_2_) in a diverse cohort of subjects undergoing invasive exercise testing for exercise intolerance and early‐stage PH. Rest gas exchange parameters were predictive of the presence or absence of PVD. Moreover, in both study and validation cohorts, none of the subjects with noncardiac exercise limitations had both abnormal rest gas exchange parameters. In evaluating patients with undefined dyspnea in the ambulatory setting, this simple noninvasive resting tool may allow practitioners to screen patients for PVD, and possibly exclude those who are deconditioned and would garner minimal benefit from invasive testing.

Early recognition of PH phenotypes with a physiological method of exercise is essential and more sensitive than saline loading.^28^, ^29^ With the standardization of exercise hemodynamics, we utilized the widely accepted standards of pressure–flow relationships and workload‐dependent abnormalities,^17^, ^23^, ^24^, ^30^ and identified three distinct clinically relevant groups. In addition to phenotyping PH, exercise testing provides insights into the burden of limitations from varying mechanisms, including cardiac, pulmonary vascular, chronotropic incompetence, and peripheral muscular disease.^31^, ^32^ In this study, both PVD and PVH groups had differentiable cardiopulmonary hemodynamics. However, they were equally impaired, with limitations of poor stroke volume augmentation and blunted heart rate response.

The abnormalities of gas exchange parameters (*V*_E_/*V*CO_2_ and ETCO_2_) revealed a similar trend at rest and with exercise, which provided an opportunity to explore the clinical utility of rest *V*_E_/*V*CO_2_ and ETCO_2_ in discriminating PH phenotypes. Moreover, the predictive ability of rest gas exchange parameters to identify PVD was significant (based on ROC analysis), in comparison to other widely available noninvasive echocardiographic parameters (PASP).^8^ With this approach, the thresholds for rest *V*_E_/*V*CO_2_ (>40 mm Hg) and ETCO_2_ (<30 mm Hg) were similar to historically reported thresholds to identify moderate‐severe PH, as summarized in a comprehensive review by Arena et al.^27^ However, most studies were based on nonsimultaneous RHC and noninvasive CPET. These include studies focused on PAH^14^, ^32^, ^33^, ^34^, ^35^, ^36^, ^37^, ^38^ and heart failure with preserved and reduced ejection.^31^, ^39^, ^40^, ^41^ Taylor et al.^42^ reported a worsening trend of *V*_E_/*V*CO_2_ and ETCO_2_ in combined pre‐/postcapillary PH), in comparison to isolated postcapillary PH, among 28 subjects with left heart disease‐related PH undergoing invasive exercise hemodynamics. Overall, a strong consistent trend in *V*_E_/*V*CO_2_ and ETCO_2_ among different PH phenotypes in these studies involving subjects of different age and sex improves confidence in our findings.

While most subjects in this study (45/55) had PH at rest, significant exercise intolerance (out‐of‐proportion to suspected PH) prompted exercise testing to discriminate extracardiopulmonary limitations. Nevertheless, this unique patient cohort meets the goals of this study to investigate the clinical utility of rest gas exchange parameters to identify PVD by clinicians taking care of this “less‐sick” population. Hence, we propose that this simplified approach can be expanded to a broad cohort of patients evaluated for dyspnea in an ambulatory setting as a screening tool.

### 4.1 Limitations

This was a single‐center retrospective study with a moderate sample size. While a majority of the patients had a diagnosis based on resting hemodynamics, the study addresses a need for early identification of PVD with noninvasive gas exchange markers in a specific group of patients with exercise intolerance and dyspnea at an early disease stage. Future studies analyzing gas exchange behavior in exercise PH only are warranted. Lastly, this study protocol did not include a radial arterial line, which limits the use of particular data (arterial oxygen content: CaO_2_ and partial pressure of carbon dioxide: PaCO_2_) and limits the calculation of dead space. However, all subjects had maintained SpO_2_ > 95% with the pretest six‐minute walk test, and during the invasive exercise hemodynamic study. Hence, it does not affect the goals of this study.

## 5 CONCLUSION

In a cohort of subjects with exercise intolerance and suspected early‐stage PH, rest *V*_E_/*V*CO_2_ and ETCO_2_ can identify patients who are likely to have PVD. Such patients may benefit from a prompt invasive hemodynamic evaluation and PH vasodilator therapy. This simple‐to‐use diagnostic test can be used as a screening tool in an ambulatory setting.

## AUTHOR CONTRIBUTIONS

Farhan Raza and Naga Dharmavaram collected the data. Farhan Raza, Timothy Hess, Ravi Dhingra, James Runo, Naomi Chesler, and Marlowe Eldridge reviewed the data, performed analysis, and formulated the manuscript. Farhan Raza, Amy Chybowski, Callyn Kozitza, and Marlowe Eldridge wrote the manuscript. Supria Batra and Evelyn M. Horn collected data, performed data analysis, and wrote the manuscript section related to the validation cohort. All coauthors reviewed and approved the manuscript before submission.

## CONFLICTS OF INTEREST

The authors declare no conflicts of interest.

## Data Availability

The data that support the findings of this study are available from the corresponding author upon reasonable request.
